# Swyer-James-MacLeod syndrome with unilateral pulmonary fibrosis: a case report

**DOI:** 10.1259/bjrcr.20160105

**Published:** 2017-04-27

**Authors:** Omar Abdulla, John Cain, John Howells

**Affiliations:** Department of Radiology, Lancashire Teaching Hospitals NHS Foundation Trust, Royal Preston Hospital, Preston, UK

## Abstract

Swyer-James-MacLeod syndrome is a rare, complex disease characterized by the radiological finding of unilateral hyperlucent lung due to pulmonary oligaemia and alveolar hyperdistention as a consequence of previous obliterative bronchiolitis (bronchiolitis obliterans). Idiopathic pulmonary fibrosis is a chronic, progressive, fibrosing interstitial pneumonia of unknown cause characterized by bilateral, chronic, progressive and irreversible fibrosis limited to the lungs. We report an interesting case of Swyer-James-MacLeod syndrome affecting one lung and Idiopathic pulmonary fibrosis affecting the contralateral lung.

## Case

A 72-year-old Caucasian female patient was referred to the respiratory clinic with a 6-month history of exertional breathlessness. She gave a history of yearly chest infections, particularly in winter months. She, however, had no history of childhood respiratory problems or any significant respiratory symptoms during her adult life. She had a 30-pack-year smoking history but she quit 25 years prior to presentation. She worked as a nursing auxillary at a local hospital and had no prior asbestosis. She was on a 1 mg maintenance dose of prednisolone for polymyalgia rheumatica. Otherwise, she had no significant medical condition and was not on any regular medications. Her exercise tolerance was unlimited and she enjoyed a good health.

Her O_2_ saturation was 95% on room air and her spirometry showed obstructive pattern with FEV1 1.14L (55%), FVC 2.09L (83%) and FEV1/FVC ratio 54%.

**Figure 1.**
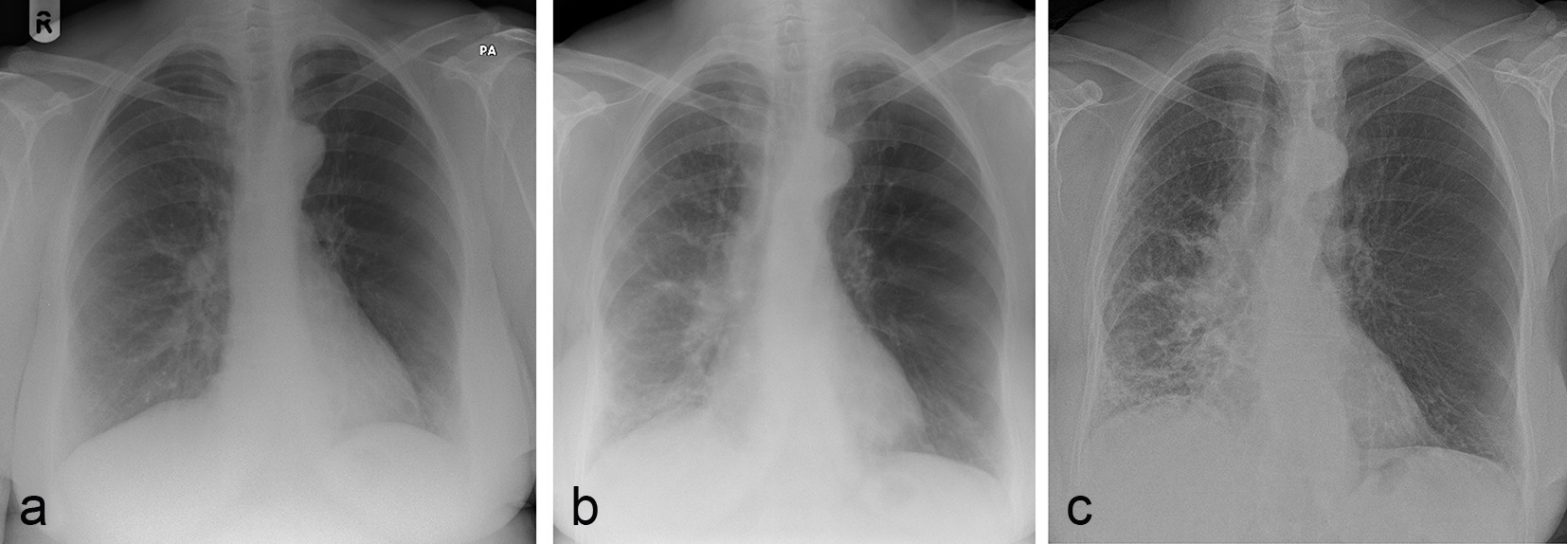
Serial CXRs (a, b and c) showing hyperlucent left hemithorax with progressive reticular shadowing and volume loss of the right middle and lower lobes.

An initial chest radiograph 2 years prior to presentation suggested an increased radiolucency of the left lung but this was not investigated further (Figure 1a). Serial CXRs 2 years later showed a relatively unchanged appearance of the left hemithorax with progressively increased opacification in the periphery of the middle and lower zones, and accompanying reduction in lung volume, on the right side (Figure 1b,c).

The patient underwent a contrast-enhanced CT of the chest in view of the repeated chest infections and lack of complete resolution to rule out the possibility of an adenocarcinoma *in situ* or endobronchial lesion, respectively. Cross-sectional imaging was also carried out to assess the possibility of an underlying bronchiectasis in the presence of the recurrent chest infections and to further assess the interstitial changes seen on the CXR on the right side.

**Figure 2.**
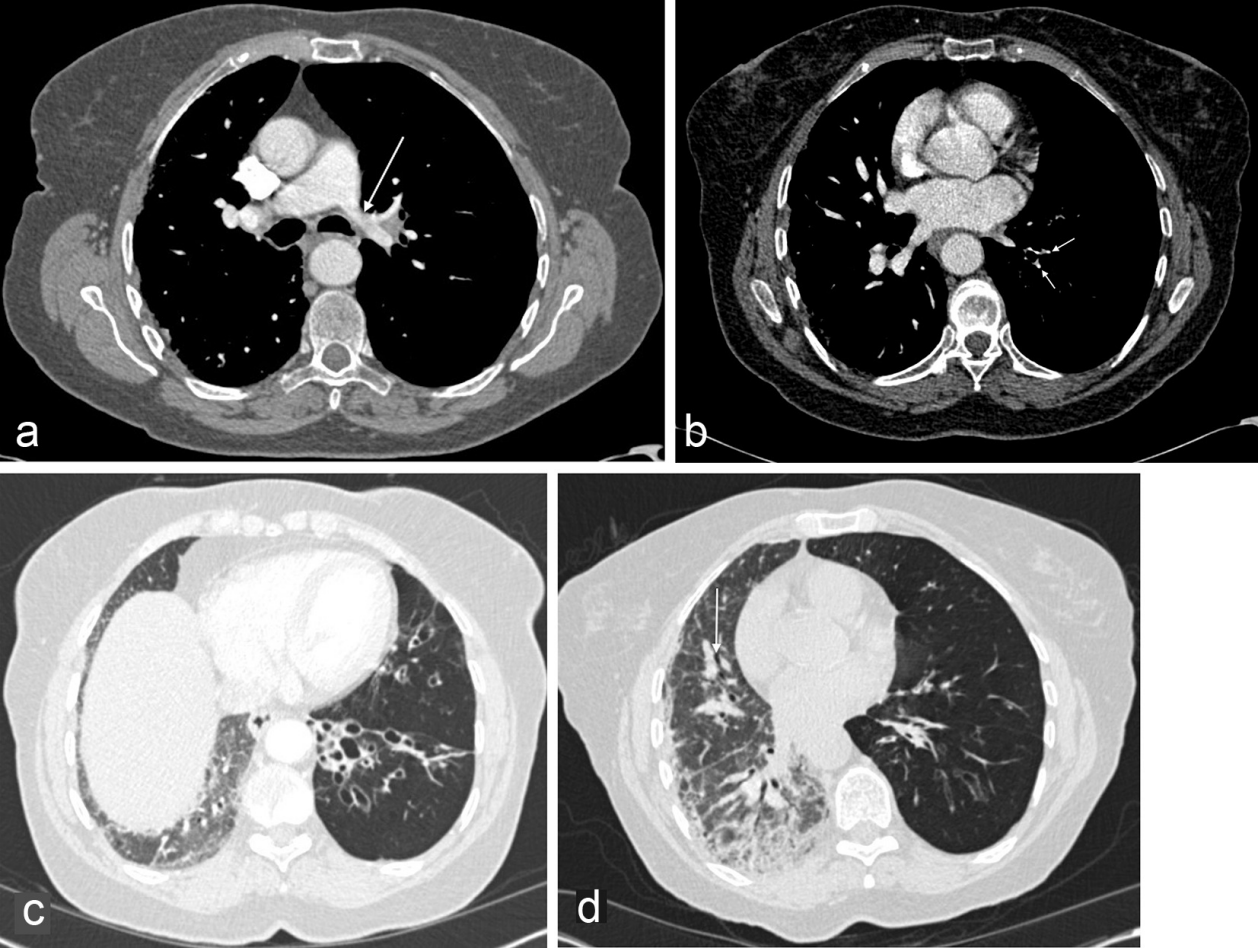
Contrast-enhanced CT of the chest shows hypoplastic left pulmonary artery (a, arrow), reduction in the number and size of the peripheral pulmonary artery branches on the left (b, arrows), translucent left lung with cystic bronchiactasis of the lingula and left lower lobe (c, d). The right lung shows volume loss, extensive subpleural reticulation with lower lobe predilection (c, d) and traction bronchiectasis (d, arrow).

**Figure 3.**
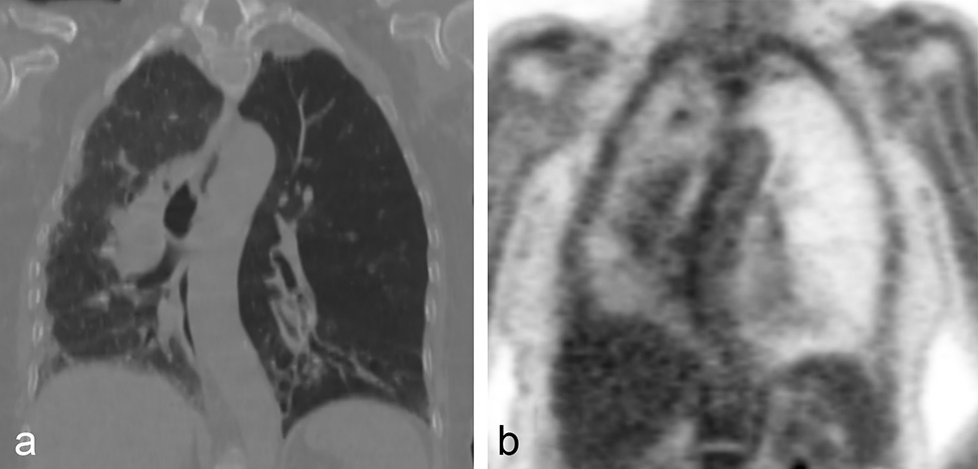
PET CT for investigation of an unrelated pathology demonstrates oligaemia of the left hemithorax with generalized reduction in FDG uptake (a and b).

Her CT showed hypoplastic left pulmonary artery (Figure 2a; arrow), attenuated peripheral pulmonary artery branches on the left (Figure 2b: arrows), hyperinflated left lung with air trapping and cystic bronchiectasis of the lingua and left lower lobe (Figure 2c,d). Features were in keeping with the diagnosis of Swyer-James-MacLeod syndrome (SJMS). Interestingly, the right lung was small in size with evidence of subpleural reticulation, traction bronchiectasis and ground-glass opacification suggesting an underlying pulmonary fibrosis (Figure 2c,d). The oligaemia of the left lung is shown as reduced FDG uptake (Figure 3) on PET CT, which was done to investigate a different pathology.

Sputum culture grew pseudomonas species and bronchoalveolar lavage from the right lung showed mixed cellularity with 22% eosinophils and a 36% neutrophils.

## Discussion

Swyer and James first described in 1953 a unilateral translucent lung with diminished vascular markings in a young child,^1^ followed by MacLeod in 1954, who published a series of nine patients describing similar findings.^2^

SJMS is a rare entity characterized by the radiological finding of unilateral hyperluscent or translucent lung. The primary pathology is the development of obliterative bronchiolitis following various insults, most commonly childhood infections. Other aetiologic factors include medications, radiation therapy and foreign body aspiration. Reduced lung capacity for ventilation causes secondary reflex vasoconstriction leading to attenuation of peripheral pulmonary vasculature with fewer and narrower vessels, and hypoplasia of the ipsilateral pulmonary artery due to reduced blood flow. Lung oligaemia in SJMS is a combination of diminished pulmonary capillary beds secondary to interalveolar septal fibrosis, mechanical resistance by overinflated terminal airspaces and reflex vasoconstriction to minimize the ventilation-perfusion mismatch.^3^ The combination of air trapping and oligaemia leads to the translucent appearance of the affected lung on chest radiograph. The bronchial system is unobstructed. The reported incidence of SJMS is 0.01% on reviewing 17,450 consecutive chest radiographs.^4^ The differential diagnoses of unilateral hyperlucent hemithorax are listed in Table 1.^5^

**Table 1.** Differential diagnoses of unilateral hyperlucent hemithorax

Pulmonary parenchyma	Unilateral emphysematous or bullous disease, pneumatocele
Airway	Foreign body aspiration, Swyer-James syndrome, congenital lobar emphysema, bronchial atresia, endobronchial mass, extrinsic bronchial compression, bronchial endotracheal-tube intubation
Blood vessels	Pulmonary agenesis, pulmonary hypoplasia, proximal interruption of the pulmonary artery, scimitar syndrome, unilateral pulmonary venous atresia, unilateral congenital pulmonary lymphangiectasia, unilateral massive central pulmonary embolism
Pleural space	Anterior pneumothorax, contralateral layering pleural effusion, diaphragmatic hernia or rupture
Chest wall	Poland syndrome, scoliosis
External (technical)	Patient rotation, lateral decentering

Idiopathic pulmonary fibrosis (IPF) is the most common form of fibrotic lung disease and is characterized by chronic, progressive and irreversible fibrosis that typically causes reticular septal thickening (with tendency to involve the subpleural and posterior basal regions), honeycombing and traction bronchiectasis. Ground-glass infiltrate and consolidation can also be seen but they are less specific. Although the aetiology of the disease is unknown, there are a number of recognized risk factors which play a role in the development of this condition in genetically predisposed individuals, including smoking, infections and environmental exposure to metal and wood dusts.

The estimated incidence of IPF in the UK is 4.6 per 10,000 person-years.^6^

Although the bronchoalveolar lavage showed high oesinophil count, there was no suspicion of oesinophilic pneumonia given the clinical and radiological pictures. Bronchoalveolar lavage has a limited role in the diagnosis of IPF. A finding of raised neutrophils (>5%) and eosinophils (>2%) is characteristic but not diagnostic of IPF.^7^

We believe this is an interesting case to report for several reasons. Firstly, to our knowledge, there are no reported cases in the literature which describe a combination of SJMS in one lung and IPF in the other one. As IPF characteristically involve both lungs (although asymmetrically), it seems that the presence of SJMS is “protective” against future development of IPF in the affected lung. We have no exact explanation of this but we propose few answers based on our understanding of the pathobiology of both conditions. In SJMS, the inflammatory response causes submucosal fibrosis of the peripheral bronchi and bronchioles with luminal irregularity and occlusion leading to hyperinflation of the distal air sacs, interalveolar septal fibrosis, alveolar destruction and obliteration of the distal airways. In IPF, wound healing involves the interstitial and alveolar spaces of the lung. The repetitive alveolar epithelial damage provokes a dysfunctional repair process whereby an uncontrolled and disorganized proliferation of myofibroblasts occurs^8^ which release an abnormal extracelluar matrix protein that causes a gradual distortion of the pulmonary architecture by honeycomb formation.^8^ The myofibroblasts are mainly derived from the differentiation of resident lung mesenchymal cells (differentiating into collagen-secreting and extracellular matrix producing cells) and epithelial-mesenchymal transition where local epithelial cells adopt fibroblast-like characteristics.^9^ Clearly, the myofibroblasts play a central role in the fibrotic process and therefore, alveolar and interstitial damage by SJMS may affect the production of these primitive cells.

A third source of the myofibroblasts is derived from the extravasation of the circulating fibrocytes, which originate from the bone marrow and differentiate in the tissue into myofibroblasts.^10^ This third source can, in theory, be affected by the oligaemia related to SJMS (see above). The latter factor can also affect the inflammatory stage of healing and recruitment of the inflammatory cells (neutrophils, oesinophils, lymphocytes and macrophages), which are known to play a part in promoting wound healing and contributing to fibrosis.

In summary, the myofibroblasts play a central role in the pathogenesis of IPF. The sources of the myofibroblasts are the epithelial cells, resident mesenchymal cells and circulating fibrocytes. SJMS seems to “deplete” the lung from these sources by affecting the epithelial and mesenchymal precursors with obliterative scarring, and also by oligaemia which reduces the extravasation of the circulating myofibroblasts.

The second reason why we believe this case is worth reporting is the fact that the diagnosis of SJMS is made in our case after the age of 70 years. SJMS is usually diagnosed in childhood in symptomatic children but if the pulmonary damage is minimal and the patient is asymptomatic or minimally symptomatic, the diagnosis can be delayed until adulthood where it is made when symptoms progress, like in our case, or when imaging is carried out for another reason. Although unusual, many cases of adult diagnosis SJMS were reported in literature; Aşker et al^11^, for example, reported an adult diagnosis for a 60-year-old patient. Therefore, advanced age should not deter clinicians from suspecting this diagnosis when history, symptoms and imaging features are compatible.

Thirdly, the original description of SJMS included a small or a normal size of the affected lung.^2^ Large-sized hyperlucent lung is a rare variant reported in the literature.^12^ In our case, it is difficult to ascertain whether the affected lung is larger than the contralateral one, or indeed is normal-sized or smaller in comparison with a significantly smaller contralateral side affected by pulmonary fibrosis.

Fourthly, the availability of PET CT imaging highlights the pulmonary oligaemia.

## Learning points

1. Coexistence of SJMS with other primary pulmonary disease is possible (in this case the coexistence of SJMS with IPF). Making these diagnoses has huge implications on patient’s treatment and prognosis.
2. It is possible that the presence of SJMS has a “protective” effect against future development of IPF.
3. When the damage is minimal and the patient is asymptomatic or mildly symptomatic, the diagnosis of SJMS can be delayed until adulthood.
4. SJMS is one of the causes of a unilateral hyperlucent or translucent hemithorax. However, other diagnoses should be kept in mind when faced with such presentation (Table 1).
5. Although plain radiographs can show suggestive pulmonary changes for both diseases, CT plays a crucial role in making the final diagnosis.

## Consent

Written informed consent for the case to be published (including images, case history and data) was obtained from the patient(s) for publication of this case report, including accompanying images.
